# Supplementary material for: Alteration of Excitation/Inhibition Imbalance in the Hippocampus and Amygdala of Drug-Resistant Epilepsy Patients Treated with Acute Vagus Nerve Stimulation
Source: Brain Sci. 2023 Jun 21;13(7):976. doi: 10.3390/brainsci13070976 (PMC10377456; doi:10.3390/brainsci13070976)
Supplement: Supplementary file 1 [file brainsci-13-00976-s001.zip › brainsci-2413470-Supplementary.pdf]

brain sciences

MDPI

Article

Alteration of Excitation/Inhibition Imbalance in the Hippocampus and Amygdala of Drug-Resistant Epilepsy Patients Treated with Acute Vagus Nerve Stimulation

Qian Yi Ooi [1], Xiaoya Qin [1,2], Yuan Yuan [1,2,*], Xiaobin Zhang [3,*], Yi Yao [3,4], Hongwei Hao [1] and Luming Li [1,2,5]

1 National Engineering Research Center of Neuromodulation, School of Aerospace Engineering, Tsinghua University, Beijing 100084, China
2 Precision Medicine and Healthcare Research Center, Tsinghua-Berkeley Shenzhen Institute, Tsinghua University, Shenzhen 518071, China
3 Department of Functional Neurosurgery, Xiamen Humanity Hospital, Fujian Medical University, Fuzhou 350005, China
4 Surgery Division, Epilepsy Center, Shenzhen Children's Hospital, Shenzhen 518038, China
5 IDG/McGovern Institute for Brain Research, Tsinghua University, Beijing 100084, China
* Correspondence: yyuan918@163.com (Y.Y.); zxb8989@126.com (X.Z.)

check for updates

Citation: Ooi, Q.Y.; Qin, X.; Yuan, Y.; Zhang, X.; Yao, Y.; Hao, H.; Li, L. Alteration of Excitation/Inhibition Imbalance in the Hippocampus and Amygdala of Drug-Resistant Epilepsy Patients Treated with Acute Vagus Nerve Stimulation. *Brain Sci.* **2023**, *13*, 976. https://doi.org/10.3390/brainsci13070976

Academic Editor: Firstname Lastname

Received: 8 May 2023
Revised: 9 June 2023
Accepted: 16 June 2023
Published: 21 June 2023

Copyright: © 2023 by the authors. Licensee MDPI, Basel, Switzerland. This article is an open access article distributed under the terms and conditions of the Creative Commons Attribution (CC BY) license (https://creativecommons.org/licenses/by/4.0/).

Table S1. Clinical characteristics of the patients.

Subject	Patient 1	Patient 2	Patient 3	Patient 4	Patient 5
Gender	Male	Male	Male	Male	Male
Age at VNS implantation	8 years	5 years 11 months	3 years 9 months	4 years	5 years 7 months
Age at SEEG assessment	9 year 4 months	8 years 11 months	8 years 2 months	5 years 6 months	6 years 10 months
Age at epilepsy onset	6 months	2 years 4 months	1 year 4 months	1 month	1 year 1 month
Etiology	Missense mutation of DEPDC5 gene; Lennox-gastaut syndrome	SCN7A gene mutation	Sequelae of encephalitis	Infantile spasm; Dysplasia of the left frontal and insular cortex	MCD
MRI	MRI negative	Bilateral cortical dysplasia	Bilateral brain atrophy	Left hemispheric volume reduction; Loss of gray-white matter differentiation in the left frontal lobe	Left temporo-parieto-occipital focal cortical dysplasia
Seizure type	Tonic, spasm, myoclonic	Spasm, tonic, focal, mixed	Myoclonic, clonic, partial	Tonic, spasm, partial	Spasm, tonic, complex partial
Seizure onset	Left hemisphere	Left frontal lobe, middle of anterior fronto-temporal lobe, parietal lobe	Multiple lesions in both hemispheres, especially in the left hemisphere	Left hemisphere	Left temporo-parieto-occipital lobe
VNS outcome	Seizure frequency was reduced by about 50%; Status epilepticus happened more frequently when VNS device was turned off	Seizure frequency was reduced by about 50%	Seizure frequency was decreased by 90% at the sixth month of VNS therapy but began to increase gradually from the ninth month; Current seizure frequency was about 30% lower than that before VNS therapy	Frequency of subtle seizure was decreased by less than 25%; Duration of GTCS was shortened from 10 min to 3 min	Seizure frequency was reduced by less than 25%; Walking and language were slightly improved

VNS, vagus nerve stimulation; SEEG, stereo-electroencephalography; MRI, magnetic resonance imaging; MCD, Malformations of cortical development; GTCS, generalized tonic-clonic seizures; RFTC, radiofrequency thermocoagulation

Table S2. Clinical characteristics of the patients. (Continued)

Subject	Patient 6	Patient 7	Patient 8	Patient 9	Patient 10
Gender	Male	Male	Female	Male	Male
Age at VNS implantation	1 year 6 months	3 years 11 months	4 years 9 months	8 years 2 months	4 years 2 months
Age at SEEG assessment	2 years 4 months	6 years 6 months	5 years 2 months	10 years 8 months	5 years 4 months
Age at epilepsy onset	2 years 4 months	1 year	4 months	5 years	3 years
Etiology	Sequelae of acute necrotizing encephalopathy	COL4A1 gene mutation; MCD	Infantile spasm; CACNA1E gene mutation	Hypoglycemic encephalopathy; ulegyria in bilateral	Viral encephalitis
MRI	Bilateral brain atrophy and bilateral hemispheric multifocal brain scar	Abnormal shape of left frontal lobe and right parietal lobe, polycerebellar gyrus deformity with cerebral fissure deformity	MRI negative	Encephalopathy; Bilateral ulegyria	MRI negative
Seizure type	Tonic-clonic, myoclonic	Partial, spasm	Spasm in clusters or isolation	Partial	Tonic, complex partial
Seizure onset	Multiple lesions in both hemispheres	Multiple lesions in both hemispheres	Multiple lesions in both hemispheres, especially in temporal lobe	Multiple lesions in both hemispheres	Bilateral fronto-temporo-insular lobe
VNS outcome	No improvement in seizure frequency	No improvement in seizure frequency	No improvement in seizure frequency; Slight improvement in cognition	No improvement in seizure frequency	No improvement in seizure frequency; Consciousness was recovered faster after GTCS than that before VNS therapy; Mouth twitching was no longer present

VNS, vagus nerve stimulation; SEEG, stereo-electroencephalography; MRI, magnetic resonance imaging; MCD, Malformations of cortical development; GTCS, generalized tonic-clonic seizures
