# Supplementary figures and images for: Alteration of Excitation/Inhibition Imbalance in the Hippocampus and Amygdala of Drug-Resistant Epilepsy Patients Treated with Acute Vagus Nerve Stimulation
Source: Brain Sci. 2023 Jun 21;13(7):976. doi: 10.3390/brainsci13070976 (PMC10377456; doi:10.3390/brainsci13070976)

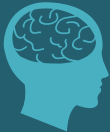

*brain  
sciences*

Supplement: Supplementary file 1 [file brainsci-13-00976-s001.zip › Definitions/brainsci-logo-eps-converted-to.pdf]

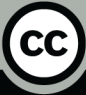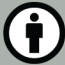

BY

Supplement: Supplementary file 1 [file brainsci-13-00976-s001.zip › Definitions/logo-ccby-eps-converted-to.pdf]

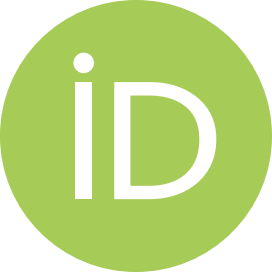

Supplement: Supplementary file 1 [file brainsci-13-00976-s001.zip › Definitions/logo-orcid.pdf]

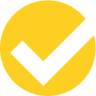

check for  
updates

Supplement: Supplementary file 1 [file brainsci-13-00976-s001.zip › Definitions/logo-updates-eps-converted-to.pdf]

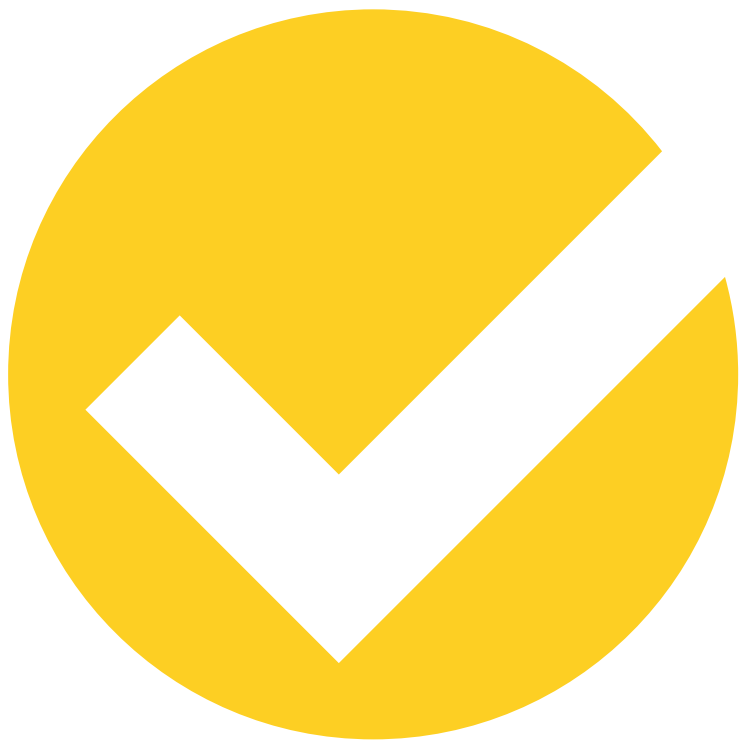

check for  
updates

Supplement: Supplementary file 1 [file brainsci-13-00976-s001.zip › Definitions/logo-updates.pdf]
